# Induced hypernatremia in patients with moderate-to-severe ARDS: a randomized controlled study

**DOI:** 10.1186/s40635-021-00399-3

**Published:** 2021-07-05

**Authors:** Shailesh Bihari, Shivesh Prakash, Dani L. Dixon, Elena Cavallaro, Andrew D. Bersten

**Affiliations:** 1 Department of ICCU, Flinders Medical Centre, Bedford Park, SA 5042 Australia; 2 College of Medicine and Public Health, Flinders University, Adelaide, SA 5001 Australia

**Keywords:** ARDS, Hypernatremia, Hyperosmolarity, Lung injury score, Mechanical Ventilation, Randomized control study

## Abstract

**Background:**

Induced hypernatremia and hyperosmolarity is protective in animal models of lung injury. We hypothesized that increasing and maintaining plasma sodium between 145 and 150 mmol/l in patients with moderate-to-severe ARDS would be safe and will reduce lung injury. This was a prospective randomized feasibility study in moderate-to-severe ARDS, comparing standard care with intravenous hypertonic saline to achieve and maintain plasma sodium between 145 and 150 mmol/l for 7 days (HTS group). Both groups of patients were managed with lung protective ventilation and conservative fluid management. The primary outcome was 1-point reduction in lung injury score (LIS) or successful extubation by day 7.

**Results:**

Forty patients were randomized with 20 in each group. Baseline characteristics of severity of illness were well balanced. Patients in the HTS group had higher plasma sodium levels during the first 7 days after randomization when compared with the control group (*p* = 0.04). Seventy five percent (15/20) of patients in the HTS group were extubated or had ≥ 1-point reduction in LIS compared with 35% (7/20) in the control group (*p* = 0.02). There was also a decrease in length of mechanical ventilation and hospital length of stay in the HTS group.

**Conclusion:**

We have shown clinical improvement in patients with moderate-to-severe ARDS following induced hypernatremia, suggesting that administration of hypertonic saline is a safe and feasible intervention in patients with moderate-to-severe ARDS. This suggests progress to a phase II study.

*Clinical Trial Registration* Australian and New Zealand Clinical Trials Registry (ACTRN12615001282572)

**Supplementary Information:**

The online version contains supplementary material available at 10.1186/s40635-021-00399-3.

## Background

ARDS patients have a high mortality rate [1]. Despite improvements in care of ARDS patients, such as the use of lung protective, volume and pressure-limited mechanical ventilation, none of the several evaluated therapeutic strategies have consistently improved outcomes in ARDS. In addition, many interventions, such as prone positioning, inhaled nitric oxide and use of extra corporeal techniques, are resource and labour intensive [2].

Both induced hypernatremia and hyperosmolarity are protective in animal models of lung injury [3–6] with possible strengthening of the lung endothelial barrier [4, 5] and increased type 1 alveolar epithelial cell repair [7]. Increasing plasma sodium with administration of hypertonic sodium is commonly performed in patients with traumatic brain injury and with raised intra cranial pressure [8, 9], and is a relatively simple and inexpensive bedside intervention in critically ill patients. However, it is not known if this simple intervention is feasible, safe and effective in patients with ARDS. Current evidence suggests that an increase in plasma sodium and osmolarity is associated with worse patient outcomes [10–15]. However, this was not the case in patients admitted to the ICU with respiratory diagnoses, or those with possible lung injury and hypoxia requiring mechanical ventilation [16, 17], suggesting a possible protective effect.

The therapeutic potential of hypertonic saline is demonstrated by its efficacy when used following initiation of injury, in various forms or both direct and indirect lung injury (ischemia–reperfusion and pancreatitis-induced acute lung injury [18, 19], oleic acid injury, intraperitoneal lipopolysaccharide, intra-tracheal lipopolysaccharide and acid aspiration-induced lung injury), through both reduced inflammation and enhanced resolution [3, 20–22]. This provided the basic and physiological rationale for its use in this study.

In this proof of concept study, we test the hypothesis that increasing and maintaining plasma sodium between 145 and 150 mmol/l in patients with moderate-to-severe ARDS [23] is feasible and will reduce the severity of lung injury. Evidence supporting this hypothesis would provide a convincing rationale for conducting a large phase II study powered to detect a difference in patient centred outcomes.

## Methods

This was a prospective randomized proof of concept single-centre study. Our study protocol was approved by the institutional human research ethics committee (Southern Adelaide Clinical Human Research Ethics Committee—HREC/15/SAC/50 (55.15)) and prospective written consent was obtained from all participants, or the next of kin, as appropriate. The trial was registered with the Australian and New Zealand Clinical Trials Registry (ACTRN12615001282572 Registered, 25^th^ Nov 2015, https://www.anzctr.org.au/Trial/Registration/TrialReview.aspx?id=369658). Patients admitted to a single tertiary ICU were prospectively recruited between December 2015 and February 2020, inclusive.

Following informed consent, we enrolled ICU patients who were ≥ 16 years of age, intubated, and within 48 h of a diagnosis of moderate/severe ARDS ARDS (PaO_2_/FiO_2_ ≤ 200 mmHg, PEEP ≥ 5 cm H_2_O, bilateral opacities on chest X-ray and respiratory failure not fully explained by cardiac failure or fluid overload) [23]. We excluded patients with active bronchospasm or a history of significant chronic obstructive pulmonary disease or asthma, moderate and severe traumatic brain injury, the presence of an intracranial pressure monitor, or any medical condition associated with a clinical suspicion of raised intracranial pressure, lack of consent (treating physician or next of kin), inevitable and imminent death, pregnancy, those receiving ECMO, or involvement in other prospective clinical studies.

These patients were block randomized with a block size of 4 patients to the hypertonic saline group (HTS) or control group. Patients in the HTS group were managed with administration of intravenous 20% saline to maintain plasma sodium between 145 and 150 mmol/l until extubation, or for a maximum period of 7 days, with use of a pre-defined protocol (Additional file 1: Figure S1). Both groups were managed with lung protective ventilaton and the use of conservative fluid management therapy at the discretion of the treating medical team, using a modified version of the ARDSnet protocol as used in the LOVS study [24, 25].

The study intervention was continued for a maximum of 7 days, or discontinued earlier if extubated, if the treating clinician felt that it was in the patient’s best interest to cease the study intervention, or if consent was withdrawn. Following cessation of the study intervention, patient management continued according to the standard local ICU protocol. The extubation protocol included daily assessments of patients' readiness to wean when the oxygen requirement was less than 40% and was at the discretion of intensive care unit clinicians.

As this was a pilot and proof of concept study, we used a prespecified and previously examined primary end point [26] which was a 1-point reduction in lung injury score (LIS) or successful extubation by day 7. Multiple secondary outcomes which included ventilator free days at 28 days, chest radiological (radiographic assessment of lung edema (RALE) scores [27], use of rescue therapy (inhaled NO, ECMO, prone positioning), LIS at day 7, length of mechanical ventilation, ICU and hospital length of stay, were also examined.

Demographic details were collected along with ICU admission diagnosis, day of stay in ICU at enrolment and measures of severity of illness [acute physiology and chronic health evaluation (APACHE) version III, and sequential organ failure assessment (SOFA) [28] score. The presence of comorbidity was assessed by the Charlson comorbidity index. Pre-randomisation data, such as cumulative fluid balance, length of mechanical ventilation, ventilatory parameters, and blood gases, were collected. Daily data collection following randomisation included blood gases, plasma sodium and chloride (highest and lowest), renal function, ventilator parameters, LIS, cumulative fluid balance (difference between the fluid administered and fluid loss) and the use of rescue therapies (inhaled NO, ECMO, Prone positioning). Daily administered sodium levels were also estimated by the previously published values [29].

Additional consent was taken for collection of bloods and brochoalveolar lavage (BAL) in these patients. Consented patients underwent a mini-BAL using 60 ml of sterile saline instilled into the subsegmental middle lobe or lingula bronchus, while under sedation and mechanically ventilated in the ICU, at day 0 (admission to the ICU) and day 3. Parallel blood samples were also obtained from all participants at the time of BAL. Samples were stored at 4 °C for less than 1 h before processing. BAL was centrifuged at 150*g* for 5 min at 4 °C to obtain the cell pellet. BAL supernatant was aliquoted and stored at −80 °C until assay, as below. Blood samples were centrifuged at 1000*g* for 10 min at 4 °C before being aliquoted and stored at − 80 °C until analysis, as below. Based on the previous studies [3–6], BAL and plasma were blindly and randomly assayed for angiopoietin (Ang)-2, Ang-1, interleukin (IL)-6 and tumour necrosis factor (TNF)-α using double antibody sandwich enzyme-linked immunosorbent assays (ELISA) by commercially available kits (R&D Systems, Minneapolis, MN), according to the manufacturer’s instructions. BAL supernatant was also analysed for total soluble protein (Micro BCA protein Assay Kit, Thermo Fisher Scientific, IL).

Statistical analyses were performed using PASW 26.0 software (SPSS Inc, Chicago, IL). All data were analysed as intention-to-treat. The sample size calculation and hence separation between study groups was assumed to be in line with the previous study by Meduri et al. [26]. A sample size of 18 patients per group was calculated at a power of 80% and alpha of 0.05. To allow for contingencies, 20 subjects per group were enrolled. Data are reported as means with SD, or median with IQR, as appropriate for the distribution of each variable. Data were tested for normality by Shapiro–Wilk’s test and normalised by log transformation where necessary. The groups were compared with an independent sample *t* test or Mann–Whitney *U* test, as appropriate. Differences between variables over time were analysed by a repeated-measures analysis of variance (ANOVA) and the effect of group was analysed as an interaction effect. BAL and plasma mediator baseline differences between groups were examined by independent samples *t* test and temporal data by linear mixed modelling as an interaction effect. BAL and plasma mediators are expressed as mean ± SEM. A *p *value ≤ 0.05 was used as the level of reportable significance.

## Results

**Fig. 1:**
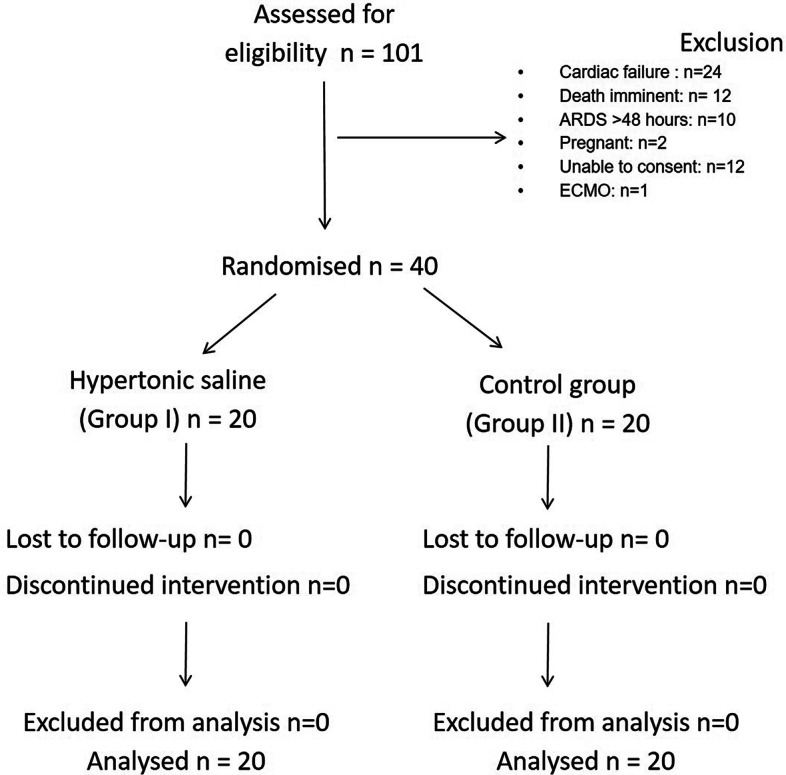
Consort diagram of patients included in the study

One hundred and one patients were assessed for eligibility during the study period. After exclusions based on the study criteria, 40 patients were randomized in the study with 20 in each group (Fig. 1).

Baseline characteristics of severity of illness were well balanced between the groups (Table 1). There were also no differences between the group’s baseline blood gases or ventilator parameters (Table 1).

**Table 1:** Baseline characteristics of patients included in the study

	Hypertonic saline (*n* = 20)	Control (*n* = 20)
Age (years)	59.5 (48.0–77.0)	66.5 (59.0–74.5)
Male (*n*,%)	13 (65%)	13 (65%)
Height (cm)	168 (165–180)	170 (168–177)
Weight (kg)	80 (68–84)	80 (70–90)
APACHE II	20 (15–22)	20 (17–22)
APACHE III	74 (40–80)	78 (54–87)
APACHE III ROD	0.25 (0.06–0.40)	0.27 (0.08–0.51)
CCI	3 (0–5)	2 (0–4)
SOFA score	9 (6–10)	11 (5–13)
Time since intubation before randomization (hours)	12.0 (2.5–16)	8 (3.0–20)
CFB (ml)	1220 (281–2340)	899 (200–1722)
LIS	3.0 (2.8–3.4)	3.0 (2.5–3.2)
Oxygenation index (OI)	12.4 (11.6–14.8)	12.7 (9.6–13.4)
ARDS Pulmonary (*n*,%)	13 (65%)	14 (70%)
Driving pressure (cm H_2_0)	12 (10–13)	12 (8–13)
PEEP (cm H_2_0)	15 (12–16)	15 (13–16)
PaO_2_/FiO_2_	132 (97–154)	140 (126–156)
PaCO_2_ (mmHg)	58 (42–69)	61 (51–69)
pH	7.31 (7.21–7.41)	7.29 (7.18–7.33)

Data presented as Median (IQR)

*APACHE* acute physiology and chronic health evaluation score, *ROD* risk of death, *CCI* Charlson Comorbidity Index, *SOFA* sequential organ failure assessment score, *CFB* cumulative fluid balance, *LIS* lung injury score, *OI* Oxygenation index

**Fig. 2:**
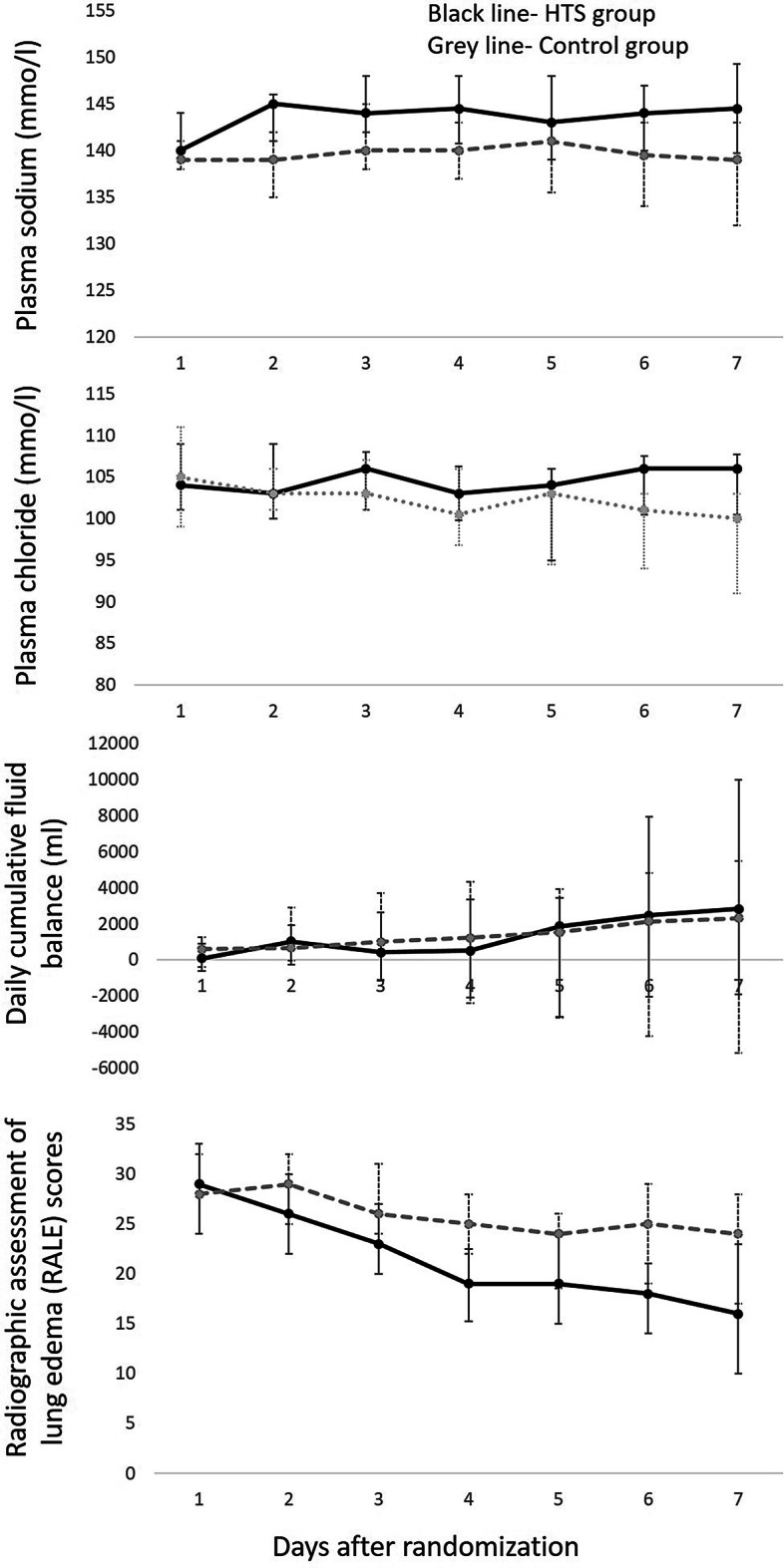
Highest daily plasma sodium, chloride levels, cumulative fluid balance and radiographic assessment of lung edema (RALE) score in the study participants during the first 7 days of the study. There was a difference in the daily sodium and chloride levels (*p* = 0.04 and 0.03, respectively), but no difference between the cumulative fluid balances (*p* = 0.48) between the groups. There was a difference in the daily RALE score (*p* = 0.03) between the groups during the study period. Data presented as median and IQR over days

Patients in the HTS group had higher plasma sodium and chloride levels during the first 7 days after randomization when compared with the control group (*p* = 0.04 and 0.03, respectively), but there was no difference in their daily cumulative fluid balance during the first 7 days (*p* = 0.48) (Fig. 2). There was no difference in their urea (*p* = 0.51) and creatinine (*p* = 0.66) levels during the study period (Additional file 1: Figure S2).

In the HTS group, 75% (15/20) of patients were extubated or had ≥ 1-point reduction in LIS compared to 35% (7/20) in the control group (*p* = 0.02). There was also a decrease in the length of mechanical ventilation and hospital length of stay in patients in the HTS group (Table 2). However, there was no difference in the LIS at day 7, ventilation free days at day 28 or ICU length of stay between the groups. There was no difference in the use of renal replacement therapy, diuretics or use of inhaled nitric oxide between the groups (Table 2). There was significant improvement in their radiographic assessment of lung edema (RALE) scores during the study period (*p* = 0.03) (Fig. 2).

**Table 2:** Primary and secondary outcomes in the study

	Hypertonic saline (*n* = 20)	Control (*n* = 20)	*p* value	Effect size
Primary outcome				
Extubated or with ≥ 1-point reduction in LIS by day 7	15 (75%)	7 (35%)	0.02	5.6 (1.4 to 21.8)
Extubated by day 7	12 (60%)	6 (30%)	0.11	3.5 (0.9 to 12.9)
≥1-point reduction in LIS by day 7	14 (70%)	7 (35%)	0.06	4.3 (1.1 to 16.3)
Secondary outcomes				
LIS at day 7^a^	1.8 (1.0–2.9)	2.8 (2.2–3.2)	0.11	0.9 (− 1.8 to 0.3)
VFD28^a^	23 (17–24)	18 (18–24)	0.10	3.7 (− 1.7 to 9.2)
LOMV (hours)^a^	133 (97–260)	336 (154–432)	0.04	− 112 (− 249 to − 10)
RRT (*n*,%)	7 (35%)	5 (25%)	0.73	1.6 (0.4 to 6.3)
Diuretics (*n*,%)	15 (75%)	17 (65%)	0.69	0.5 (0.1 to 2.6)
Inhaled NO (*n*,%)	11 (55%)	12 (60%)	1.00	0.8 (0.2 to 2.8)
ICU LOS (day)^a^	12.0 (6.6–16.7)	19.7 (9.9–37.6)	0.06	− 9.2 (− 18.7 to 0.3)
Hospital LOS (day)^a^	19.0 (13.6–26.8)	46.4 (32.9–54.1)	0.001	− 24.4 (− 37.5 to − 11.2)
Hospital mortality	3/20 (15%)	4/20 (20%)	1.00	0.7 (0.1 to 3.6)

*LIS* lung injury score, *VFD 28* ventilator free day at day 28, *LOMV* length of mechanical ventilation, *RRT* renal replacement therapy, *NO* nitric oxide, *LOS* length of stay

^a^Data presented as Median (IQR)

There was also no difference between the groups in their daily organ failure scores (*p* = 0.29), daily administered fluid volume (*p* = 0.20), daily urine output (*p* = 0.62) and daily administered sodium between the group (*p* = 0.46) (Additional file 1: Figures S3–S5). The estimated daily plasma osmolarity was higher in patients in the HTS group (*p* = 0.05) (Additional file 1: Figure S6).

There was no difference in the intubated patients between the groups in their daily highest PaO_2_/FiO_2_ ratio (*p* = 0.37), PaCO_2_ (*p* = 0.59), minute ventilation (*p* = 0.68), PEEP (*p* = 0.94), tidal volume (*p* = 0.42), plateau pressure (*p* = 0.29) and driving pressure (*p* = 0.21) (Additional file 1: Figures S7–S13). Data regarding modes of ventilation are provided in Additional file 1: Table S1.

**Fig. 3:**
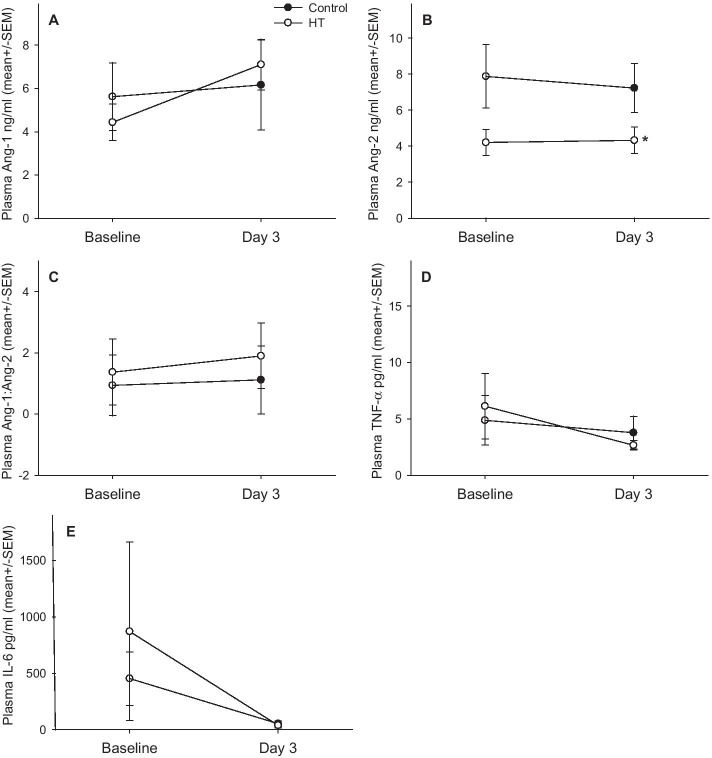

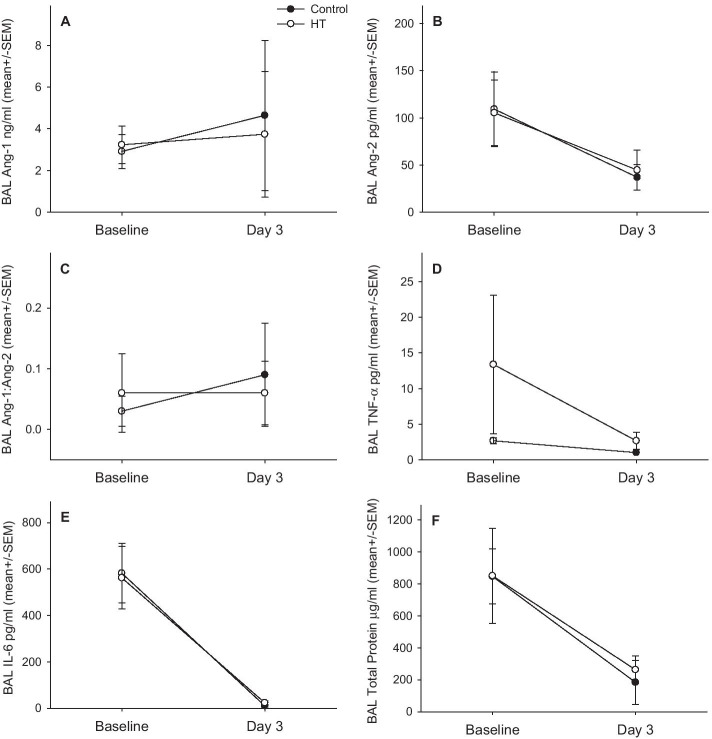
**a** Plasma Ang-1, (Group *p* = 0.936, Time *p* = 0.288, Gp*Time *p* = 0.480); Ang-2, (Group *p* = 0.010, Time *p* = 0.825, Gp*Time *p* = 0.750); Ang-1:Ang-2, (Group *p* = 0.116, Time *p* = 0.343, Gp*Time *p* = 0.639); IL-6 (Group *p* = 0.634, Time *p* = 0.158, Gp*Time *p* = 0.609); and TNF α (Group *p* = 0.972, Time *p* = 0.262, Gp*Time *p* = 0.556). **b** Bronchoalveolar lavage Ang-1, (Group *p* = 0.909, Time *p* = 0.665, Gp*Time *p* = 0.812); Ang-2, (Group *p* = 0.951, Time *p* = 0.045, Gp*Time *p* = 0.849); Ang-1:Ang-2, (Group *p* = 0.876, Time *p* = 0.376, Gp*Time *p* = 0.416); IL-6, (Group *p* = 0.972, Time p ≤ 0.001, Gp*Time *p* = 0.862); TNF α (Group *p* = 0.196, Time *p* = 0.196, Gp*Time *p* = 0.334); and total protein (Group *p* = 0.825, Time *p* = 0.005, Gp*Time *p* = 0.842) between the groups. There was no difference in these plasma and BAL biomarkers between the groups

Blood samples were available from 18 patients and BAL from 11 patients. There was no difference between the groups in plasma or BAL Ang-1, Ang-2, Ang-1:Ang-2, IL-6, TNF-α or BAL protein (Fig. 3a, b).

## Discussion

In this prospective randomized study, we found a reduction in lung injury score with earlier extubation in patients with moderate-to-severe ARDS. We also found that administration of hypertonic saline is not only a feasible intervention but appears safe in patients with moderate-to-severe ARDS, as there were no adverse effects reported including fluid balance, or other patient-related outcomes.

High plasma sodium contributes to high plasma osmolarity which can be lung protective. High plasma osmolarity has been shown to reinstate T cell function following anti-inflammatory mediator suppression in trauma [30], suppress neutrophil activation [31–33] and modify macrophage migration [34]; all of which can mitigate lung injury [31]. Hyperosmolarity increases type 1 alveolar epithelial cell repair [7], and decreases TNF-α-induced P-selectin expression which helps in remodelling of the endothelial barrier [4]. Hyperosmolar sucrose strengthens the lung endothelial barrier and enhances actin polymerization in the endothelium [4, 5]. Similarly, a brief period of vascular hyperosmolarity protects against acid-induced lung injury [6]. Conversely, hypotonicity can activate the transient receptor potential ion channels which play a critical role in lung vascular mechanotransduction leading to endothelial calcium influx, and a rise in pulmonary vascular permeability [35, 36]. In our study, these processes may have contributed to the earlier resolution of lung injury (decrease LIS) and oxygenation in the HTS group; however, we did not find changes in BAL biomarkers, and further investigation is required to elucidate the mechanisms involved.

Even though there are no prospective studies on the use of hypertonic saline in lung injury, Bulger et al. using hypertonic resuscitation showed improved ARDS-free survival among patients at risk of ARDS with massive transfusion [37]. In a randomized study, development of ARDS was less frequent when patients had received fluid loading with hypertonic saline/dextran [38]. Our study was not powered to examine any of the patient centred outcomes, but it does provide data for future studies examining these and, moreover, shows that inducing hypernatremia is both safe and feasible in ARDS patients. This study, therefore, provides credence to previous epidemiological findings of a differential effect of hypernatremia in patients with possible lung injury [16, 17].

Despite being the first study to demonstrate a decrease in lung injury with induced hypernatremia in patients with moderate-to-severe ARDS, this finding should be viewed in the context of multiple limitations. There are examples of previous ARDS research, where the findings of a small proof of concept study have not been replicated in larger trials [39] suggesting that this study warrants examination in a large study design, adequately powered for patient centred outcomes. Centre-specific practices may explain such discrepancies; for example, our ICU does not utilise prone positioning as a common intervention for patients with moderate-to-severe ARDS; however, the use of inhaled nitric oxide is more common. Hence, the application of induced hypernatremia in multiple centres with more variability of practice is important to understand the context of hypernatremia in a wider cohort of ARDS patients. We did not find any changes with renal function in our study cohort but, examination of urea and creatinine is not considered robust enough to define change in renal function; therefore, more specific markers [40] should be used in future studies to confirm this finding. We did not find any difference in cumulative fluid balance between the groups which were both managed with conservative fluid balance. However, the long-term cognitive outcomes [41] with the use of induced hypernatremia are not known. We did not had any direct quantitative measure of extra vascular lung water examined in our study, and though this was indirectly examined through the radiographic assessment of lung edema (RALE) scores, this should be included in future trials. Finally, we did not find any differences in the examined plasma and BAL sample inflammatory mediators between the groups and hence could not provide insight in the mechanism through which hypertonic saline would work on decreasing duration of invasive mechanical ventilation; moreover, there is a baseline difference in plasma Ang-2 levels; though this may indicate inherent variability, this can also be due to higher endothelial dysfunction in the control group and might have confounded our clinical findings. Future studies should examine other biomarkers associated with endothelial and epithelial lung injury exploring other mechanistic pathways. Moreover, the possible differential effects of inflammatory phenotype [42] should be examined in future larger studies.

## Conclusion

Inducing hypernatremia in moderate-to-severe ARDS patients with the use of intra-venous hypertonic saline is safe and feasible and leads to clinical improvement with earlier extubation and decrease in lung injury score. A larger phase II trial is now required to confirm these findings and to better elucidate the underlying mechanism.

## Supplementary Information

**Additional file 1.** Additional figures and tables.

## Data Availability

On request.

## Abbreviations

ARDS: Acute respiratory distress syndrome;
APACHE: Acute physiology and chronic health evaluation score
Ang: Angiopoietin
BAL: Brochoalveolar lavage
ANOVA: Analysis of variance
CCI: Charlson Comorbidity Index
ECMO: Extra corporeal membrane oxygenation
IL: Interleukin
LOMV: Length of mechanical ventilation
LOS: Length of stay
NO: Nitric oxide
SOFA: Sequential organ failure assessment score
CFB: Cumulative fluid balance
HTS: Hypertonic saline group
LIS: Lung injury score
OI: Oxygenation index
PEEP: Positive end expiratory pressure
RRT: Renal replacement therapy
ROD: Risk of death
TNF: Tumour necrosis factor
VFD 28: Ventilator free day at day 28
