# Supplementary material for: Induced hypernatremia in patients with moderate-to-severe ARDS: a randomized controlled study
Source: Intensive Care Med Exp. 2021 Jul 5;9:33. doi: 10.1186/s40635-021-00399-3 (PMC8255097; doi:10.1186/s40635-021-00399-3)
Supplement: Supplementary file 1 — Additional file 1. Additional figures and tables. [file 40635_2021_399_MOESM1_ESM.docx]

**Additional file 1: Figure S1:** Study intervention protocol used in patients randomized to the HTS group.

Patient with ARDS and plasma Na^+^ ≤145, randomized into hypertonic saline group

**YES**

**NO,**

**Na ≥150**

**NO**

Monitor Na at least once every 4 hours for at least 24 hours after 1st sample in desired range. If the target Na range sustained for 24 hours, the frequency of Na check can be decreased to once every 6 hours.

Administer 20% saline 10ml over 30 minutes through central line.

Check blood sodium (Na) levels*

Change in Na > 11 mmol/L within past 24 hours?

Is the patient’s Na level between 145-150?

**YES**

**SAFETY INTERVENTION I**

(see next page)

**SAFETY INTERVENTION II** (see next page)

**NO,**

**Na ≤145**

**Safety intervention I:** Monitor Na at least once every 2 hours for 24 hours to ensure maximum change in Na levels within 24 hours does not exceed 12 mmol/L. Treating clinician to be informed for correction using 5% dextrose if change > 12 mmol/L.

**Safety intervention 2:** If plasma sodium is ≥150 but ≤ 155 mmol/l, then it can be monitored once every 2 hours to ensure it does not exceed 155 mmol/l. If the range is still between 150 and 155 mmol/l (even after 12 hours ) or goes above 155 mmol/l (anytime) then treating clinician to be informed for correction using 5% dextrose.

**Additional file 1: Figure S2**: Highest plasma urea and creatinine levels in the study participants the first 7 days of the study. There was no difference between the groups in their daily urea and creatinine levels. Data presented as median and IQR over days.


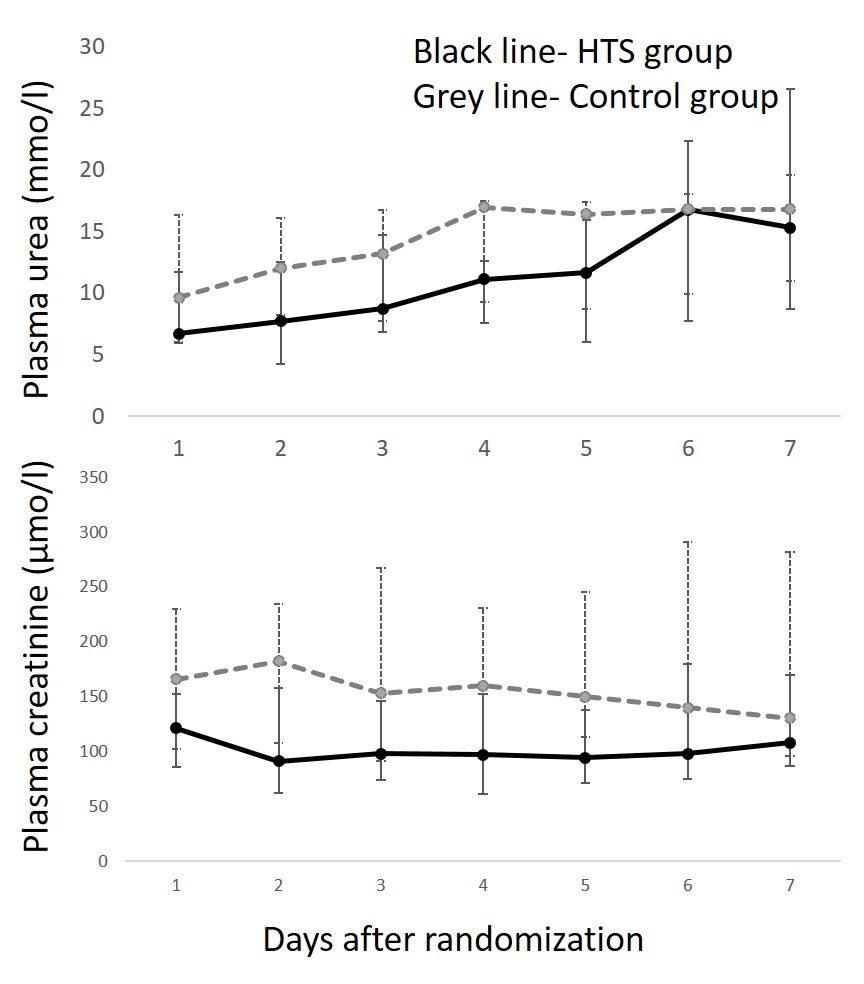


**Additional file 1: Figure S3:** Daily organ failure score (SOFA) in the study participants during the first 7 days of the study. There was no difference between the groups in daily organ failure score. Data presented as median and IQR over days.


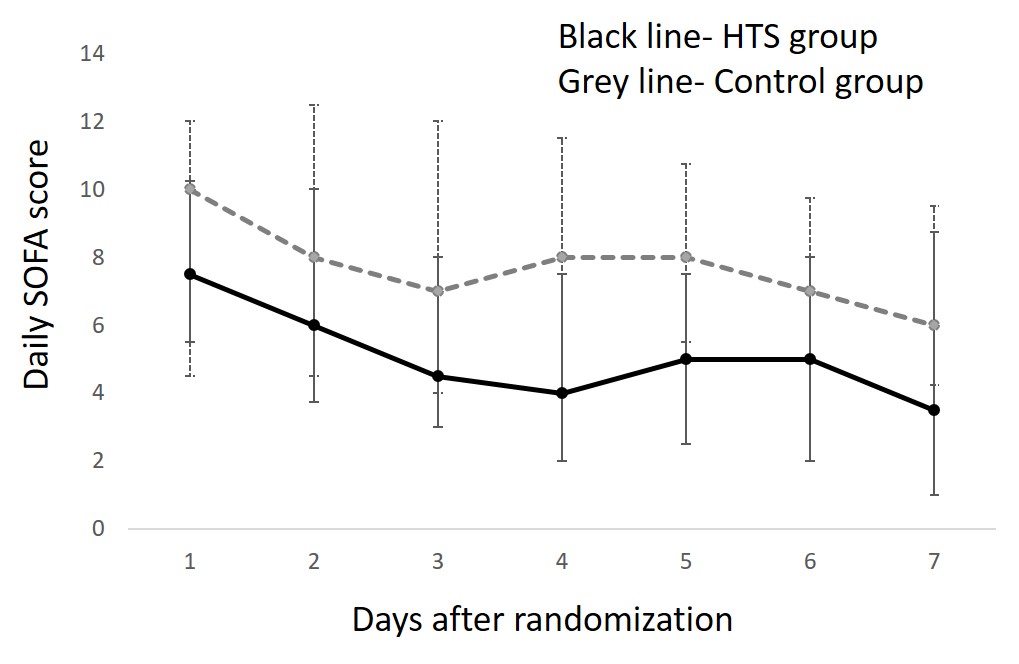


**Additional file 1: Figure S4:** Daily administered fluid volume and urine output in the study participants during the first 7 days of the study. There was no difference between the groups in daily administered fluid and urine output. Data presented as median and IQR over days.


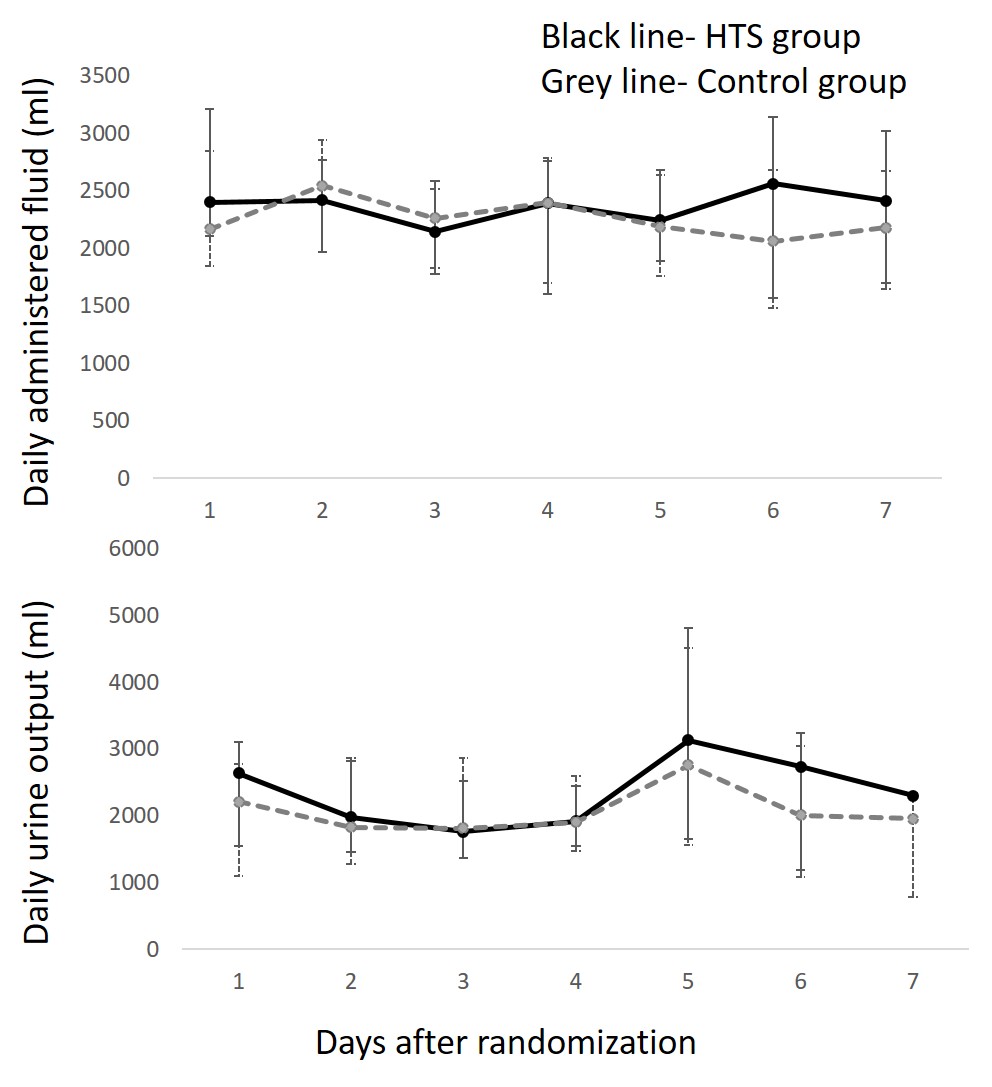


**Additional file 1: Figure S5:** Daily administered sodium in the study participants during the first 7 days of the study. There was no difference between the groups in daily administered sodium. Data presented as median and IQR over days.


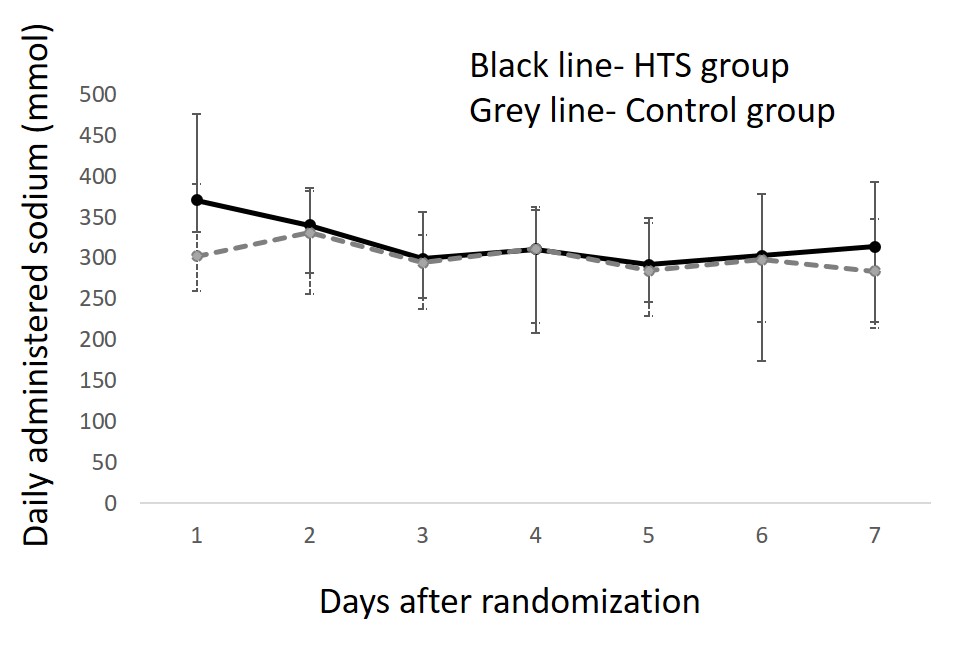


**Additional file 1: Figure S6**: Daily estimated plasma osmolarity in the study participants during the first 7 days of the study. This was higher in the HTS group during the study period. Data presented as median and IQR over days.


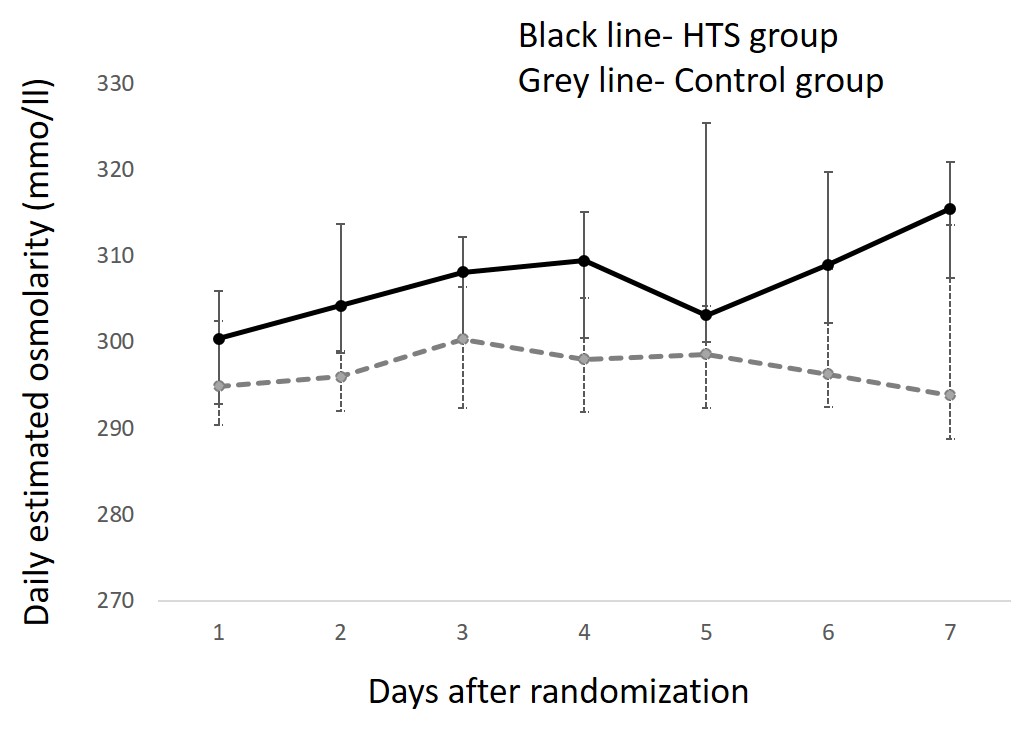


**Additional file 1: Figure S7:** Daily highest PaO_2_/FiO_2_ ratio in intubated patients during the first 7 days of the study. There was no difference between the groups in daily highest PaO_2_/FiO_2_ ratio in intubated patients. Data presented as median and IQR over days.


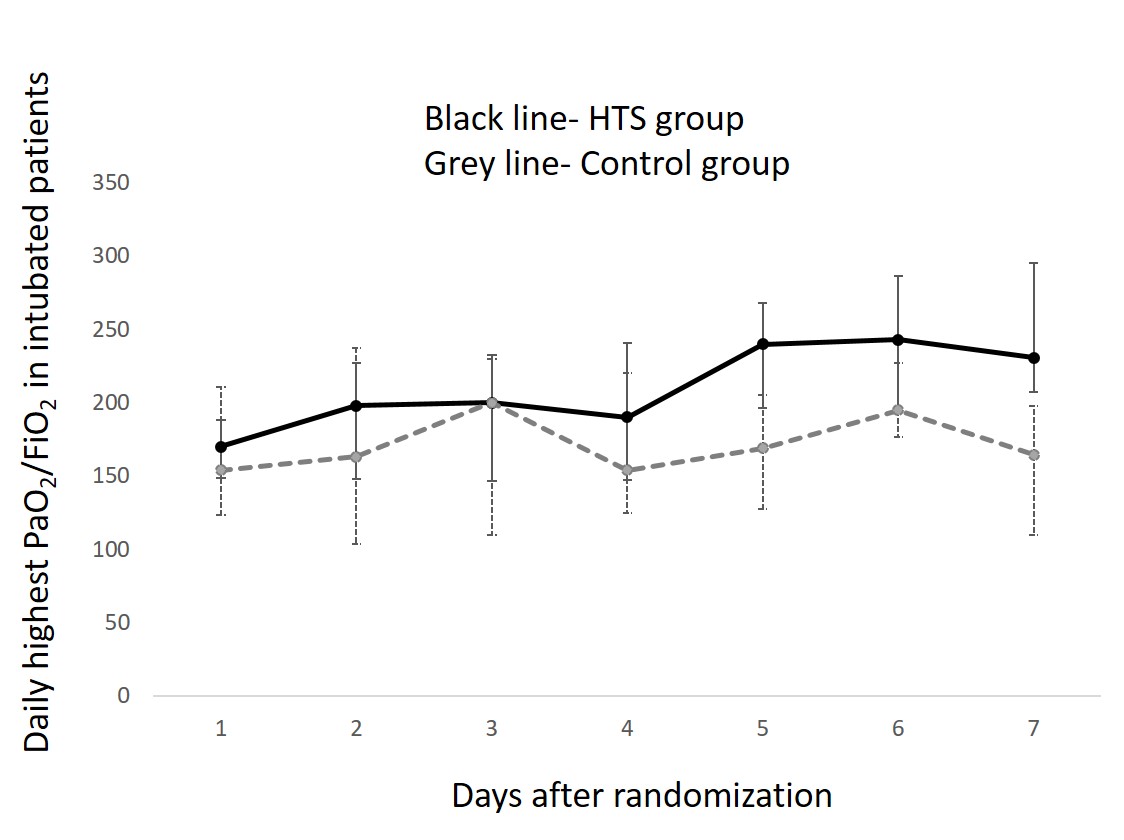


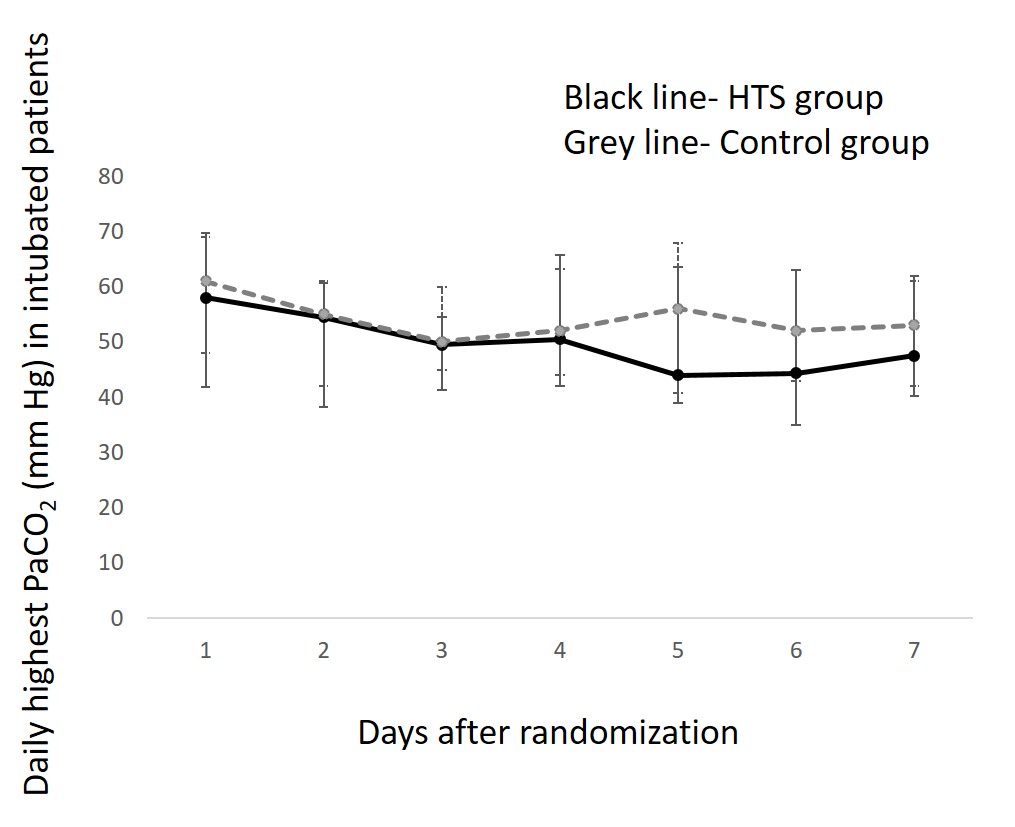
**Additional file 1: Figure S8:** Daily highest PaCO_2_ in intubated patients during the first 7 days of the study. There was no difference between the groups in daily highest PaCO_2_ in intubated patients. Data presented as median and IQR over days.

**Additional file 1: Figure S9:** Minute ventilation in intubated patients during the first 7 days of the study. There was no difference between the groups in daily minute ventilation in intubated patients. Data presented as median and IQR over days.


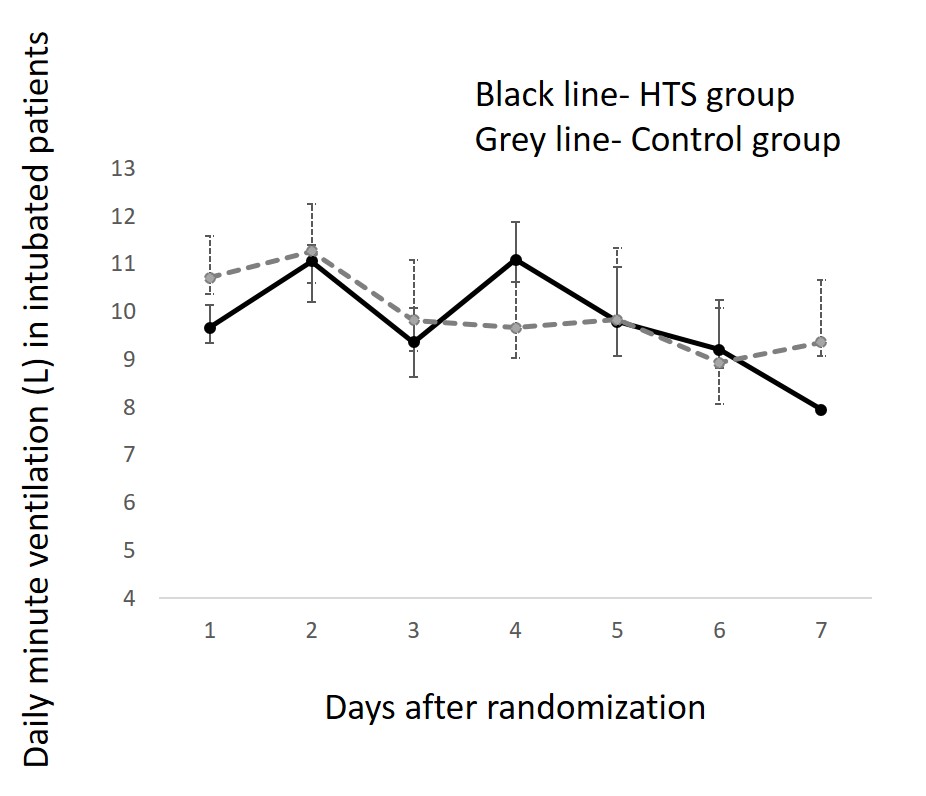


**Additional file 1: Figure S10:** Positive end expiratory pressure (PEEP) in intubated patients during the first 7 days of the study. There was no difference between the groups in daily minute ventilation in intubated patients. Data presented as median and IQR over days.


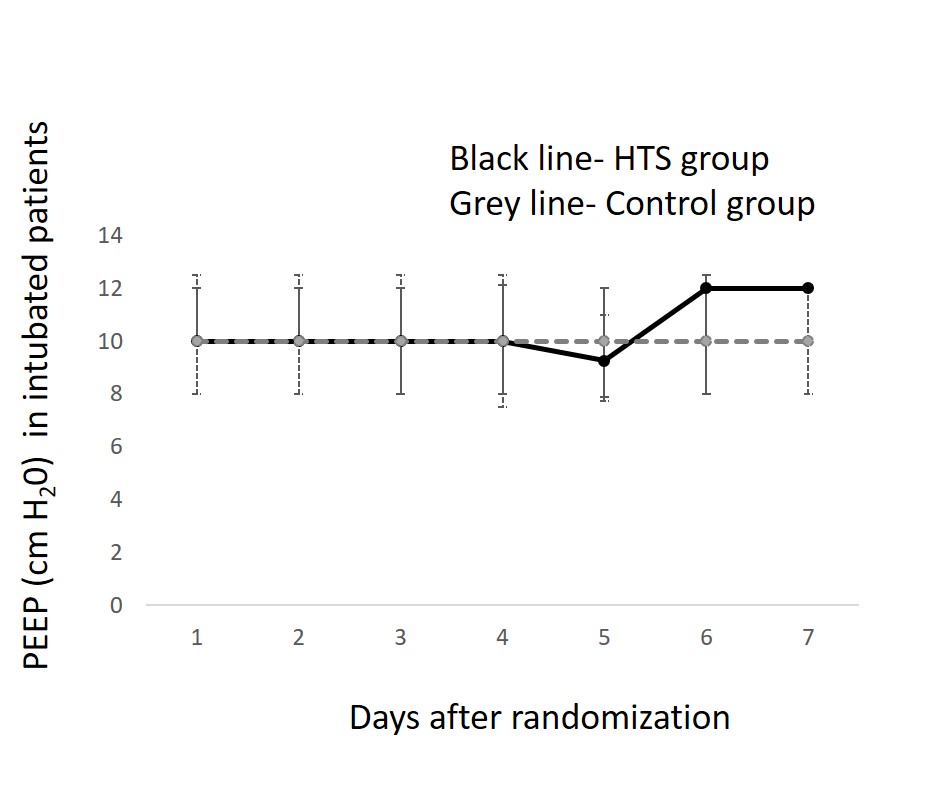


**Additional file 1: Figure S11:** Tidal volume in intubated patients during the first 7 days of the study. There was no difference between the groups in daily minute ventilation in intubated patients. Data presented as median and IQR over days.


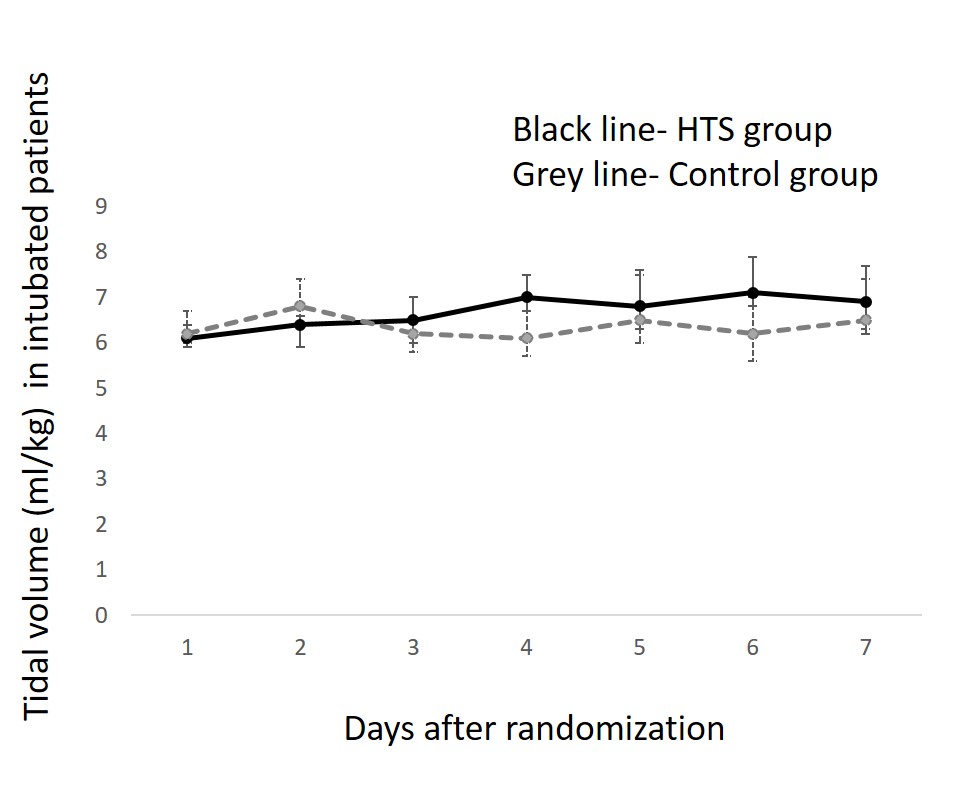


**Additional file 1: Figure S12:** Plateau pressure in intubated patients during the first 7 days of the study. There was no difference between the groups in daily minute ventilation in intubated patients. Data presented as median and IQR over days.


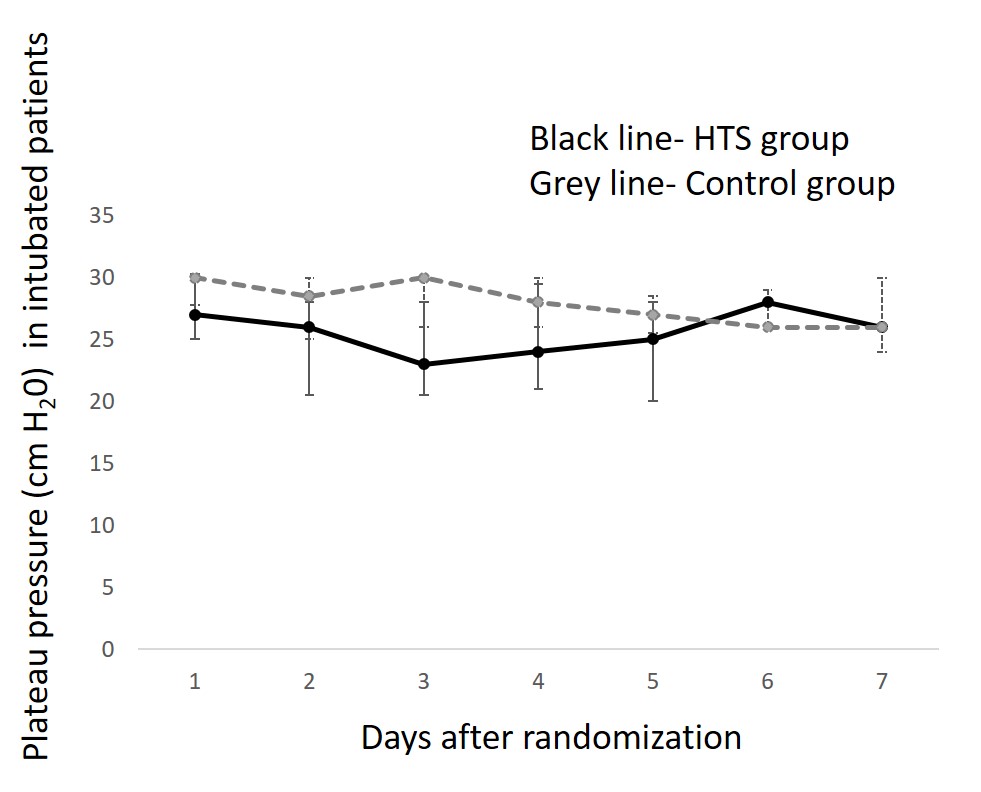


**Additional file 1: Figure S13:** Driving pressure in intubated patients during the first 7 days of the study. There was no difference between the groups in daily minute ventilation in intubated patients. Data presented as median and IQR over days.


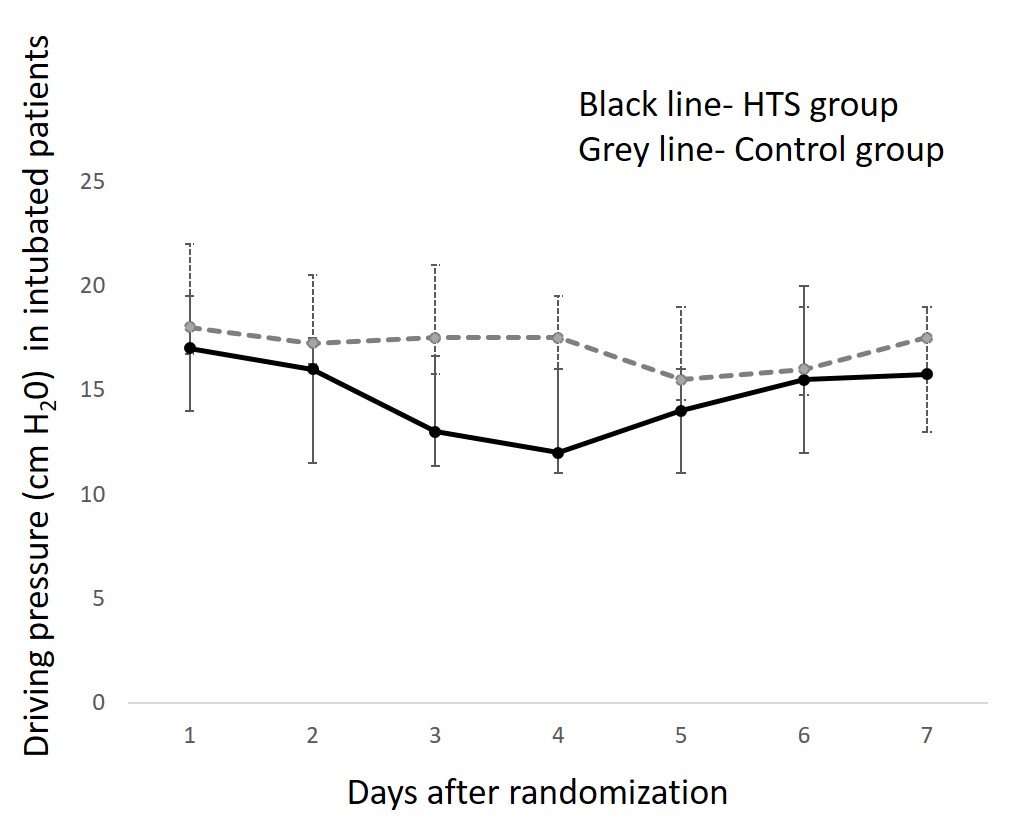


**Additional file 1: Table S1:** Modes of ventilation in both study groups during the first 7 days. Data presented as number of patients with each study group (HTS: control) on each study day.

| **Study Day** | **1** | **2** | **3** | **4** | **5** | **6** | **7** |
| --- | --- | --- | --- | --- | --- | --- | --- |
| **Volume control mode (HTS: Control)** | 20:20 | 19:18 | 16:16 | 15:14 | 10:13 | 9:13 | 7:12 |
| **Pressure support mode (HTS: Control)** | 0:0 | 1:2 | 4:2 | 2:3 | 3:4 | 1:2 | 1:2 |
| **Extubated (HTS: Control)** | 0:0 | 0:0 | 0:2 | 3:3 | 7:3 | 10:5 | 12:6 |
